# Diatomite Photonic Crystals for Facile On-Chip Chromatography and Sensing of Harmful Ingredients from Food

**DOI:** 10.3390/ma11040539

**Published:** 2018-03-31

**Authors:** Xianming Kong, Qian Yu, Erwen Li, Rui Wang, Qing Liu, Alan X. Wang

**Affiliations:** 1 College of Chemistry, Chemical Engineering and Environment Engineering, Liaoning Shihua University, Fushun 113001, Liaoning, China; xmkong@lnpu.edu.cn (X.K.); rwang@lnpu.edu.cn (R.W.); 2 School of Electrical Engineering and Computer Science, Oregon State University, Corvallis, OR 97331, USA; lie@oregonstate.edu; 3 Key Laboratory of Low Carbon Energy and Chemical Engineering, College of Chemical and Environmental Engineering, Shandong University of Science and Technology, Qingdao 266590, Shandong, China; qliu@sdust.edu.cn

**Keywords:** diatomite, photonic crystal biosilica, on-chip chromatography, SERS, food product

## Abstract

Diatomaceous earth—otherwise called diatomite—is essentially composed of hydrated biosilica with periodic nanopores. Diatomite is derived from fossilized remains of diatom frustules and possesses photonic-crystal features. In this paper, diatomite simultaneously functions as the matrix of the chromatography plate and the substrate for surface-enhanced Raman scattering (SERS), by which the photonic crystal-features could enhance the optical field intensity. The on-chip separation performance of the device was confirmed by separating and detecting industrial dye (Sudan I) in an artificial aqueous mixture containing 4-mercaptobenzoic acid (MBA), where concentrated plasmonic Au colloid was casted onto the analyte spot for SERS measurement. The plasmonic-photonic hybrid mode between the Au nanoparticles (NP) and the diatomite layer could supply nearly 10 times the increment of SERS signal (MBA) intensity compared to the common silica gel chromatography plate. Furthermore, this lab-on-a-chip photonic crystal device was employed for food safety sensing in real samples and successfully monitored histamine in salmon and tuna. This on-chip food sensor can be used as a cheap, robust, and portable sensing platform for monitoring for histamine or other harmful ingredients at trace levels in food products.

## 1. Introduction

Diatom frustules are single-celled algae that possess a biogenic silica shell with periodic nanopores on the surface [1,2]. Diatom frustules exhibit unique properties in optics, physics, and chemistry, which have attracted considerable interest for more than two centuries [3,4,5]. For example, the photonic-crystal properties of diatom can reflect vivid colors and further enhance the intensity of optical fields near the surface of the diatom [6,7]. The high surface-to-volume ratio (nearly 200 m^2^/g) makes diatoms good candidates for loading catalysts and fluid control [8,9,10,11]. In recent decades, there has been an escalating trend of fabricating ultra-high performance sensors using diatom biosilica [12]. Rorrer’s group used diatom to prepare biosensors for trinitrotoluene(TNT) sensing and good selectivity was obtained [13]. De Stefano et al. functionalized the diatom frustules with antibodies and then used them for immunoassay, in which 100 nM detection limit of protein was achieved [14]. They also deposited an Au layer on the diatom frustules and used them as a surface-enhanced Raman scattering (SERS) substrates [15]. Our group has fabricated several SERS biosensors by depositing metallic nanoparticles (NPs) near or inside the periodic nanopores of diatoms. Hybrid photonic-plasmonic modes were formed between the diatom photonic biosilica and plasmonic nanoparticles, which could enhance the local optical field near the plasmonic NPs and additional SERS enhancement was obtained [16,17,18,19,20,21]. These biosensors were employed for TNT and mouse IgG sensing, in which the sensitivity was achieved to 10^−10^ M and 10 pg/mL, respectively.

On the biosensor application side, food safety has caused growing concern because foodborne diseases account for nearly 30% of patients, even in developed countries. Scallan et al. have reported that nearly 31 kinds of major pathogens have occurred in the United States, causing nearly 10 million episodes of foodborne related illnesses and more than one thousand deaths [22]. Therefore, the identification of harmful contaminants in food products is of pivotal significance to ensure food quality. SERS spectroscopy is an advanced analytical method that is widely used for food analysis due to its high sensitivity and ability to obtain inherent information of target molecule at trace level concentrations [23,24,25]. However, the consistency and repeatability of the SERS technique is a challenge for food sensing because of the complicated components or contaminants in foodstuffs. In most cases, extraction and purification processes are needed. Thin layer chromatography (TLC) is a simple separation technology that is widely used in small molecule separation. In tandem, SERS and TLC create a powerful method for on-site identification that is simple, fast, and requires no complicated sample preprocessing [26].

In this paper, we report the progress of using diatomite photonic crystals as a robust lab-on-a-chip platform for separating and detecting harmful chemicals in complex real food samples. The diatomite used in this research has photonic crystal features as its nanoporous structure is similar to diatoms [27,28]. The TLC chips were fabricated using diatomite photonic crystal biosilica and used for identification of several kinds of harmful compounds (Sudan I and histamine) in food samples. The diatomite photonic crystal plate enhanced with plasmonic nanoparticles produced in this study could achieve nearly 10 times the increment of the intensity of SERS spectra than a normal TLC chip with silica gel layers. During testing, we successfully demonstrated on-chip detection of histamine from mixed salmon samples and furthermore, from real spoiled tuna using a TLC-SERS method without any sample pretreatment.

## 2. Experiment

### 2.1. Chemicals and Reagents

Chlorauric acid (HAuCl_4_) was obtained from Alfa Aesar (Haverhill, MA, USA). Sodium citrate (Na_3_C_6_H_5_O_7_), hexane, carboxymethyl cellulose (CMC), and ethyl acetate were supplied from Macron (Avantor, Center Valley, PA, USA). Diatomite earth (Celite209), 4-mercaptobenzoic acid (MBA), Sudan dye, and histamine were bought from Sigma-Aldrich (Saint Louis, MO, USA). The fresh salmon used in the testing was purchased from local supermarkets, and the decomposed yellow-fin tuna was kindly supplied from the Department of Food, Science, and Technology, Seafood Research and Education Center of Oregon State University. All chemical reagents are at least analytical grade. Ultrapure water (~18.2 MΩ cm) produced by a Millipore Gradient system (Millipore, Burlington, MA, USA) was used throughout the experiments.

### 2.2. Synthesis and Characterization of Au Colloid

Au colloids were synthesize using sodium citrate as the reducing and stabilizing agent in accordance with the literature [29]. Briefly, 100 mL of 1 mM aqueous chloroauric acid solution was heated to boil under vigorous stirring. After adding 4.2 mL of 1% sodium citrate, heating and stirring of the mixture continued for half an hour. Au NPs of nearly 50 nm size were obtained in the colloidal solutions.

### 2.3. Preparation of Diatomite Biosilica Plate

The diatomite plate used in the chromatography separation and SERS detection were prepared by a spin coating process. First, 11.6 g of dried diatomite powder were suspended in 20 mL of an aqueous solution of CMC (0.5%). The diatomite thin layer was deposited onto the glass slide by spin coating at 1200 rpm for 20 s. The diatomite plates were kept in shade for one day and then put in an oven at 110 °C for 3 h to enhance the interaction between the diatomite thin layer and glass slides.

### 2.4. On-Chip Separation and Detection Process

**Scheme 1:**
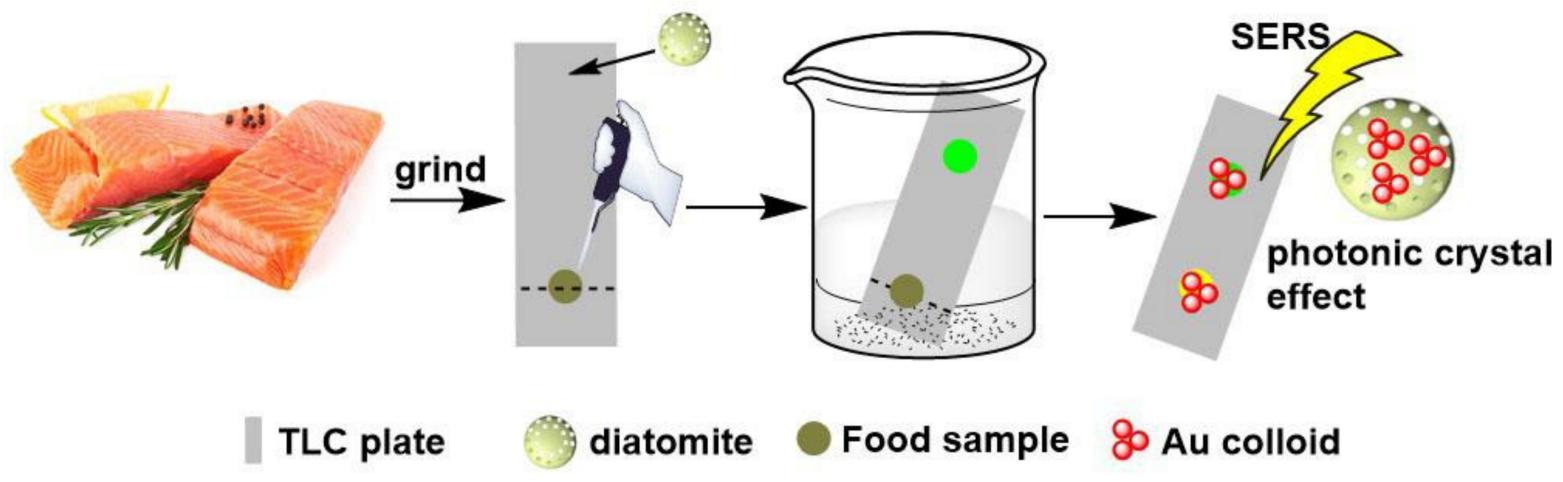
Illustration of the on-chip detection of histamine from fish by the diatomite photonic crystal chip.

The process of on-chip separation and SERS identification of hazardous chemicals in food is illustrated in Scheme 1. The liquid sample (1 μL) was spotted onto the bottom (1.2 cm) of a TLC plate. After separation of the analytes, the chromatography chips were then dried quickly with a blow drier. The separated spots corresponding to different analytes were visualized by iodine colorimetry, and then, 2 μL concentrated Au colloidal suspension was casted to each spot. The SERS measurements were performed under a confocal Raman microscope (Lab Ram HR800, Horiba JY, Kyoto, Japan) with a 785-nm excitation laser, and a 50× long working-distance objective was used. The diatomite chromatography plate was placed on the automatic XYZ stage of the Raman microscope. SERS mapping images were collected with a 10 × 10-point mapping array. The data were measured using the DuoScan module with a 2.0 μm step size and 0.5 s integration time.

### 2.5. Other Apparatuses

The UV-VIS absorption spectra of the Au colloid were collected by NanoDrop 2000 spectrometer (Thermo Scientific, Waltham, MA, USA). The colloid was put in polystyrene cuvette with a 10 mm optical path, and the scanning electron microscope (SEM) images of the diatomite, diatomite plate, and prepared Au NPs were obtained by a FEI Quanta 600 FEG SEM (FEI, Hillsboro, OR, USA). The optical images were collected by a Raman microscope (Olympus, Tokyo, Japan) with a 50× objective lens for the cross-section of the diatomite layer on the chromatography chip and a 100× objective lens for the near-field image of the single diatomite. Halogen lamps were used as the light source.

## 3. Results and Discussion

### 3.1. Characterization and Evaluation of Diatomite and the Diatomite Layer

**Figure 1:**
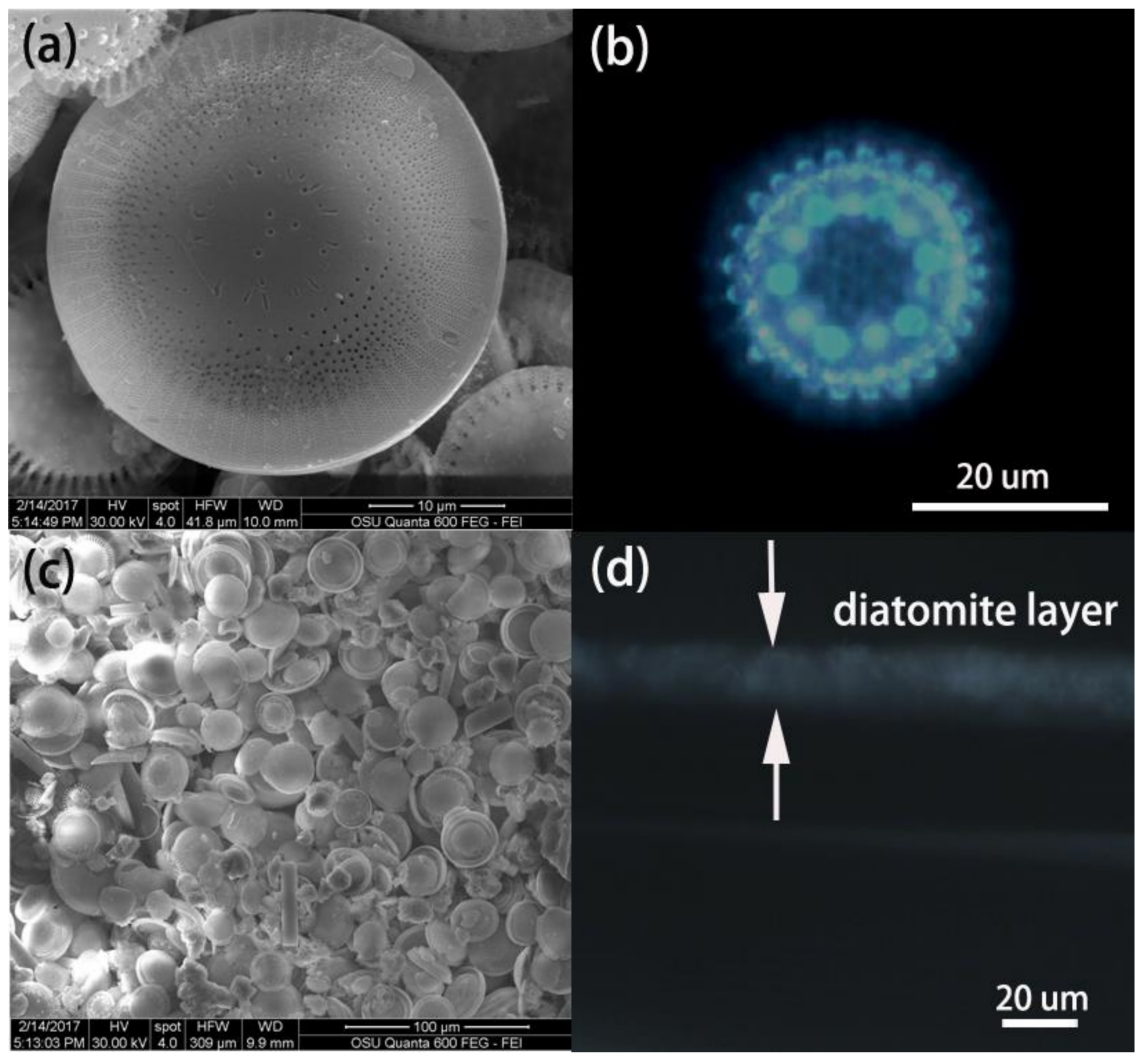
Surface morphology of diatomite biosilica with nanoporous structure (**a**), microscopic image with the diffraction pattern of diatomite (**b**), morphography of the diatomite thin layer deposited on the glass slides (**c**), and the cross-section image of the diatomite thin layer on glass (**d**).

SEM images of diatomite and the diatomite layer are shown in Figure 1. The diatomite is dish-shaped with two-dimensional (2D) periodic pores as shown in Figure 1a. The periodic porous structure of diatomite biosilica can supply photonic-crystal features, such as the guided-mode resonances (GMRs) effect [7].The optical image of diatomite biosilica was collected to confirm the photonic-crystal feature of diatomite as shown in Figure 1b. The regular optical pattern results from the high-order diffraction of the photonic crystals, which is consistent with the study from De Stefano’s group [30]. Briefly, a 100× objective lens focused onto a single diatomite frustule, and a Halogen lamp was used as the light source. The near-field image of the diatomite frustule was captured directly by the Charge-coupled device(CCD) camera of the Raman microscope. The diffraction pattern was obtained by adjusting the objective distance slightly longer than the focal length of the objective lens so that the near-field pattern right after the frustule can be imaged to the image plane (the CCD camera). The diatomite is deposited and functions as the stationary phase on the TLC chip. The surface morphology of the diatomite thin layer deposited on the glass slides are shown in Figure 1c. The size of the diatomite ranges from 10 to 30 μm. The cross-sectional thickness of the stationary phase on the glass substrate (diatomite thin layer) was measured by microscope as shown in Figure 1d. The average thickness of the diatomite layer on the glass was approximately 20 μm, which is only one-third of that of a commercial TLC chip (Figure S1).

### 3.2. Characterization of Au NPs

**Figure 2:**
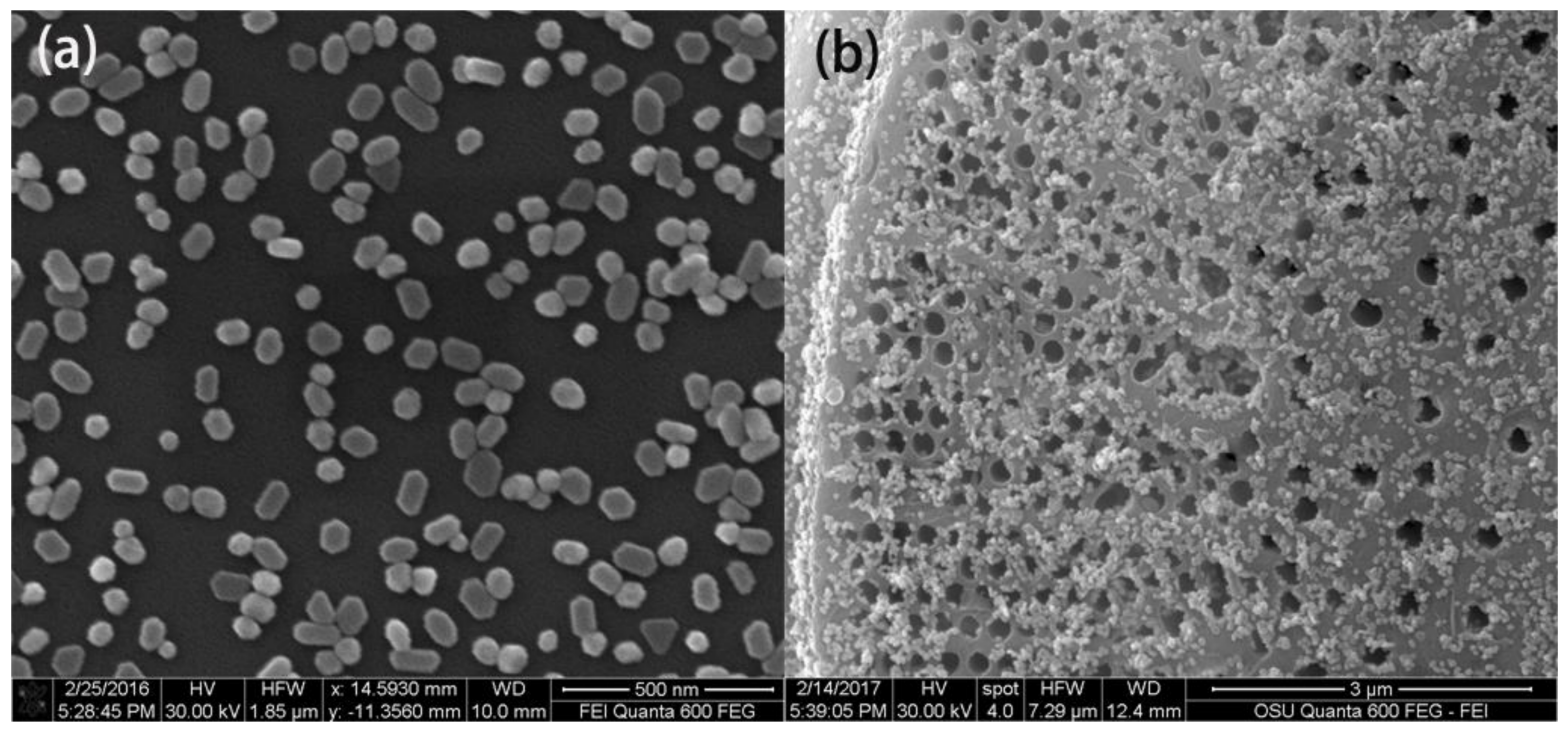
SEM image of the Au nanoparticles (NPs) used in the experiment (**a**) and the diatomite disk after casting with Au NPs (**b**).

The SEM image of prepared Au NPs with size distribution between 50–60 nm is shown in Figure 2a. From the UV-VIS spectrum of the colloid, the Au NPs’ suspension (pH at 6.5) is shown in Figure S2. The maximum absorption is located at 548 nm, which indicates that the diameters of the Au NPs were approximately 50–60 nm with relatively uniform distribution. The concentration of the Au colloidal suspension was nearly 0.8 × 10^−10^ M, which was calculated based on Lambert’s law with the molar extinction coefficient of 3.4 × 10^10^ M^−1^ cm^−1^ in accordance with the research from Navarro et al. [31]. After TLC separation, the Au NPs were deposited onto the chromatography chip and the distribution of Au NPs at the surface of the diatomite is shown in Figure 2b.

### 3.3. Optical Simulation of Plasmonic NPs on Diatomite

**Figure 3:**
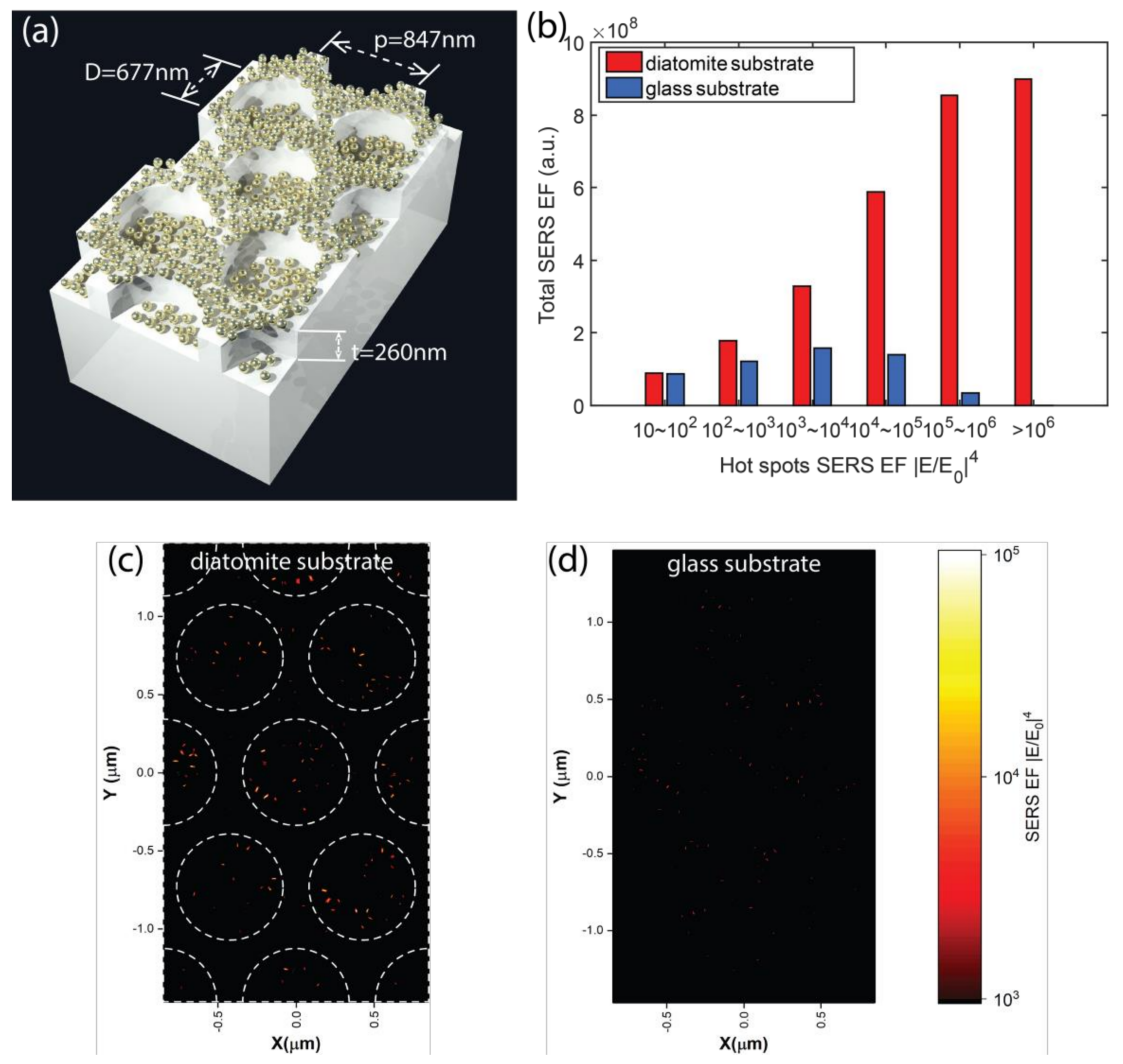
3D schematic of the finite-difference time-domain (FDTD) simulation model (**a**), statistical analysis of the total SERS enhancement factors (EF) contributed by different levels of “hot spots” on the diatomite biosilica plate and glass substrate (**b**), SERS EF distribution of the Au NPs layer on the diatomite plate (**c**) and on the glass substrate (**d**).

We performed three-dimensional (3D) finite-difference time-domain (FDTD) simulation to investigate the SERS enhancement of the diatomite biosilica plate. Figure 3a shows the 3D schematic of our simulation model. The diatomite biosilica plate was modeled as a silica 2D photonic crystal slab with a hexagonal air hole lattice placed on top of a glass (silica) substrate. The thickness of the silica photonic crystal slab t = 260 nm were obtained from the SEM measurement. The air hole distance p and air hole diameter D we used in our simulation were 847 nm and 677 nm, respectively. Such a periodic structure would give us a guided-mode resonance (GMR) at the excitation wavelength of 785 nm, which could potentially produce the largest SERS enhancement. However, it is worth noting that in real cases, diatomite silica plates may have some variation of the air hole diameter and the periodic structure. Au NPs with diameters of 70 nm were randomly distributed on the biosilica plate both inside and outside of the airholes. For comparison, we also simulated the case where the same distribution of Au NPs was placed on top of a glass substrate. Because the SERS enhancement factor (EF) is proportional to the fourth power of the local electrical field intensity [32], here we used SERS EF as the main metric, which is defined as |E/E_0_|^4^, where E_0_ is the amplitude of the incident light. The SERS signal was dominated by “hot spots” with extremely large SERS EFs generated by the localized surface plasmonic resonances (LSPRs) between the Au NP gaps. We performed a statistical analysis by integrating the SERS EFs near the Au NPs layers and clarifying the contribution from different intensity levels of “hot spots”. The results are plotted in Figure 3b. When Au NPs are placed on the diatomite biosilica plate, the LSPRs were coupled with the GMRs supported by the periodic photonic crystal biosilica plate. As a result, the overall SERS EFs were boosted, and the enhancement from diatomite biosilica plate became more prominent as we inspected stronger hot spots. Figure 3c,d plot the SERS EF distribution of the Au NPs layer on the diatomite biosilica plate and on a flat glass substrate. The color bar was chosen to show only the “hot spots” that had an EF larger than 10^3^. It is clearly shown that the density and intensity of the “hot spots” were enhanced inside the air holes of the diatomite substrate. This interesting phenomenon has been explained in our previous publication [14] by the coupling strength between the GMRs and the LSPRs.

### 3.4. SERS Analysis of the Pigment from the Artificial Mixture

**Figure 4:**
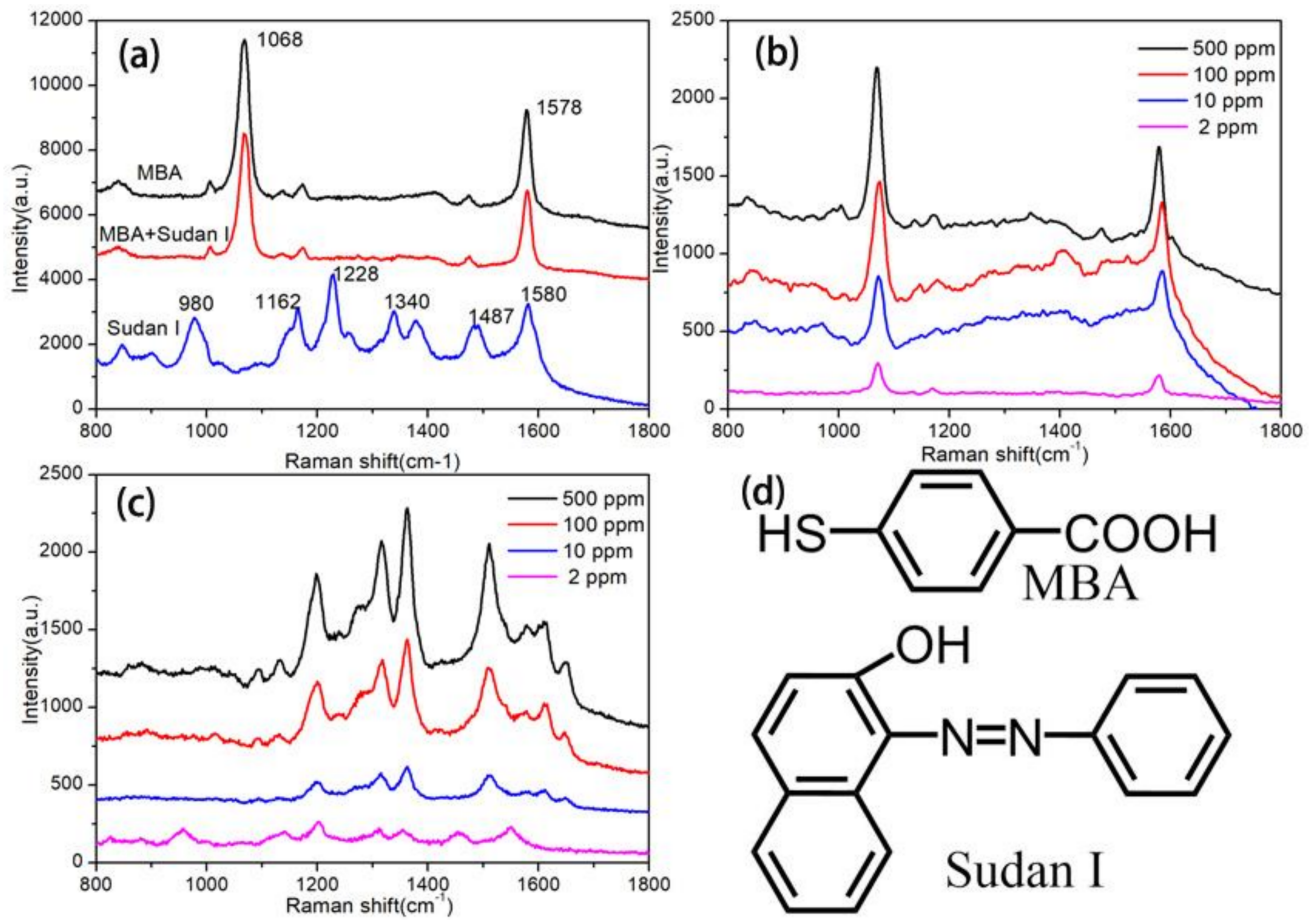
SERS spectra of the pure substances and the mixture (**a**), of 4-mercaptobenzoic acid (MBA) separated from the mixture at different concentrations (**b**), and of Sudan I separated from the mixture at different concentrations (**c**), and the molecular structure of MBA and Sudan I (**d**).

The tandem of TLC and SERS for on-chip detection was investigated for a sample produced artificially. SERS spectra were obtained for the pure substance of MBA, the pure substance of the Sudan I dye, and a mixture of MBA and Sudan I (molar ratio, 1:1). Briefly the 50 ppm of MBA, the Sudan I dye, and the mixture were added to Au colloids, respectively, and the Raman spectra were measured. The SERS spectra of the pure components and the mixture is shown in Figure 4a. The molecular structure of MBA and the Sudan I dye is presented in Figure 4d. MBA is the one of most frequently used probe analytes in SERS investigation because of the gold sulfur bond that could be generated between the S–H group and the surface of the metallic SERS substrate. For MBA, the feature Raman peaks located at 1068 and 1578 cm^−1^ are assigned to the aromatic ring breathing vibrations of MBA [33]. In the SERS spectrum of Sudan I, the Raman peaks at 1162 cm^−1^ and 1580 cm^−1^ are assigned to the vibrations of the aromatic ring and azo group of Sudan I. The strong Raman peak at 1228 cm^−1^ is assigned to several vibration modes from the phenylic group of Sudan I [34,35], which was selected as the characteristic SERS peak in the Sudan I dye analysis. From the SERS spectrum of the mixture, only characteristic peaks of MBA could be obtained because the surface of the Au NPs were totally occupied by MBA. The typical SERS method cannot read the Raman spectra information of the Sudan I dye from the mixture.

The chromatographic separation effect of the diatomite plate was investigated using the mixture (MBA and Sudan I, 1:1). Hexane and ethyl acetate (*v*/*v* = 6:1) were mixed and applied as the chromatographic solvent for separating Sudan I. The separated spots of Sudan I and MBA were visualized through red color and iodine colorimetry, respectively. Sudan I migrates at a faster rate on the TLC chip and the location was farther from the bottom edge of plate due to the fact that its molecular polarity was lower than that of MBA. The Au NPs were casted onto the different spots of the analytes. The Raman spectrum at each spot was collected and is shown in Figure 4a,b. The obvious SERS peak at 1228 cm^−1^ was assigned to the characteristic peak of Sudan I. These results indicate that the diatomite chip could be used for separating the pure component from the mixture successfully.

**Figure 5:**
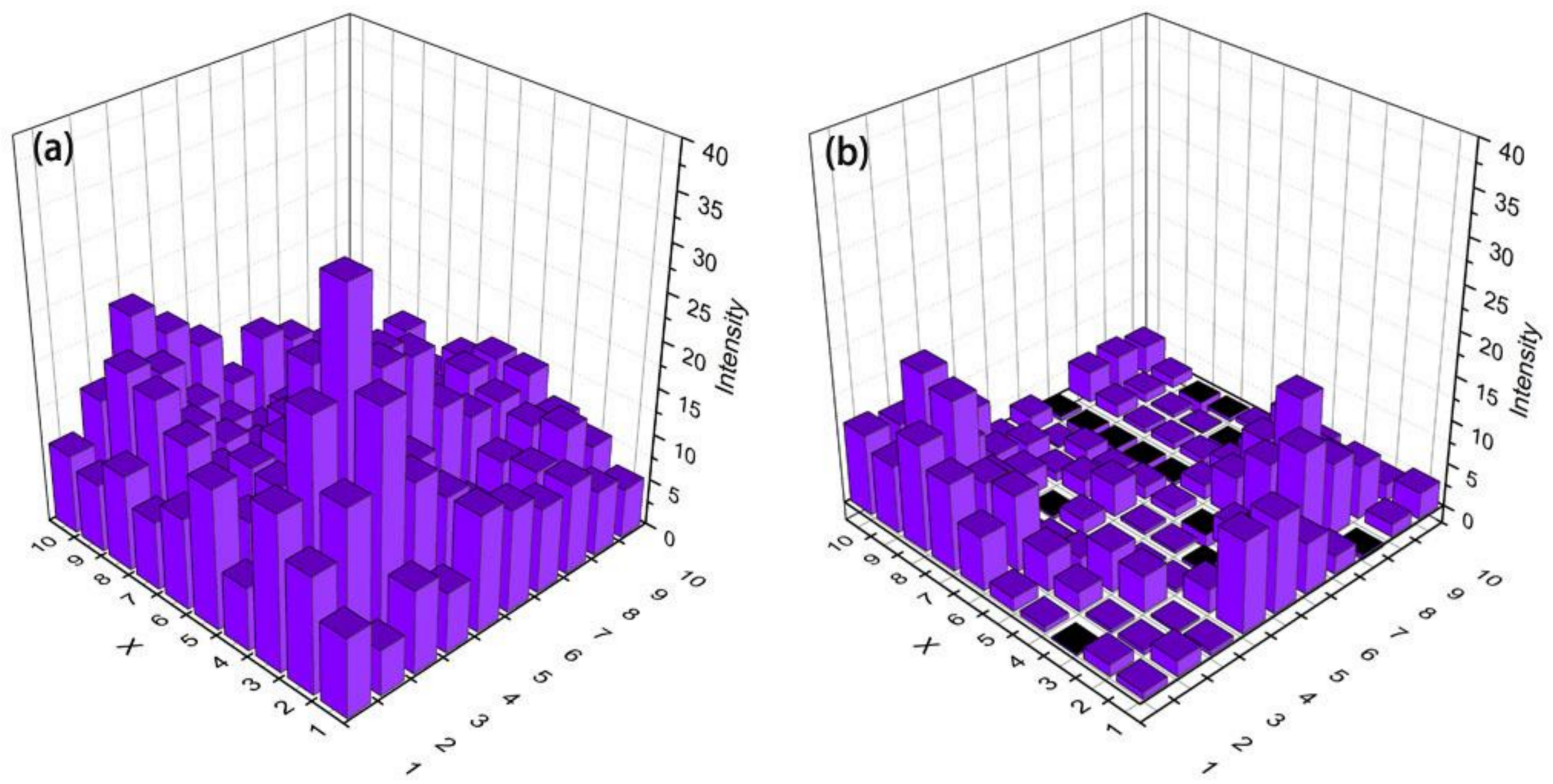
SERS mapping images of 10 ppm MBA on (**a**) The diatomite chip and (**b**) The normal TLC chip.

The uniformity of the analytes distributed on the TLC chips was visualized through Raman mapping images as shown in Figure 5. The histogram images of the SERS mapping of MBA (10 ppm) on the fabricated diatomite chip and the commercially available TLC chip are shown in Figure 5a,b. MBA provides two intense Raman peaks at 1068 and 1578 cm^−1^, which are assigned to the breathing modes of the aromatic ring of MBA. The SERS mapping data was recorded using the integrated peak intensity between 1055–1085 cm^−1^. The average value of the integrated peak intensity in Raman mapping images is 16 au and 2 au from the diatomite and the common TLC chip, respectively. The mapping results express several times the increment of SERS intensity using the diatomite chip in comparison to the normal silica gel chip. There are two reasons for this increment. On the one hand, the thickness of the stationary phase on the TLC chip is inversely proportional to the amount of analyte for the SERS measurement. Therefore, the thinner diatomite layer could provide a larger amount of analyte molecules for SERS measurement. The thickness of stationary phase on the diatomite TLC chip is nearly 20 μm, which is almost one-third of the thickness of the stationary phase on the normal TLC chip (60 μm, Figure S2). On the other hand, the periodic nanopores on the diatomite biosilica enable it with photonic-crystal features [7,36]. When plasmonic Au NPs are located near the surface of diatomite, the hybrid photonic-plasmonic resonance mode could be formed, which brings additional SERS enhancement. The uniformity of the SERS signal on the diatomite chip also better than that of the normal TLC chip as the coefficient of variation (CV = standard deviation/mean) is 0.5 on the diatomite chip and 1 on the normal TLC chip.

### 3.5. Histamine Sensing in Spoiled Fish

**Figure 6:**
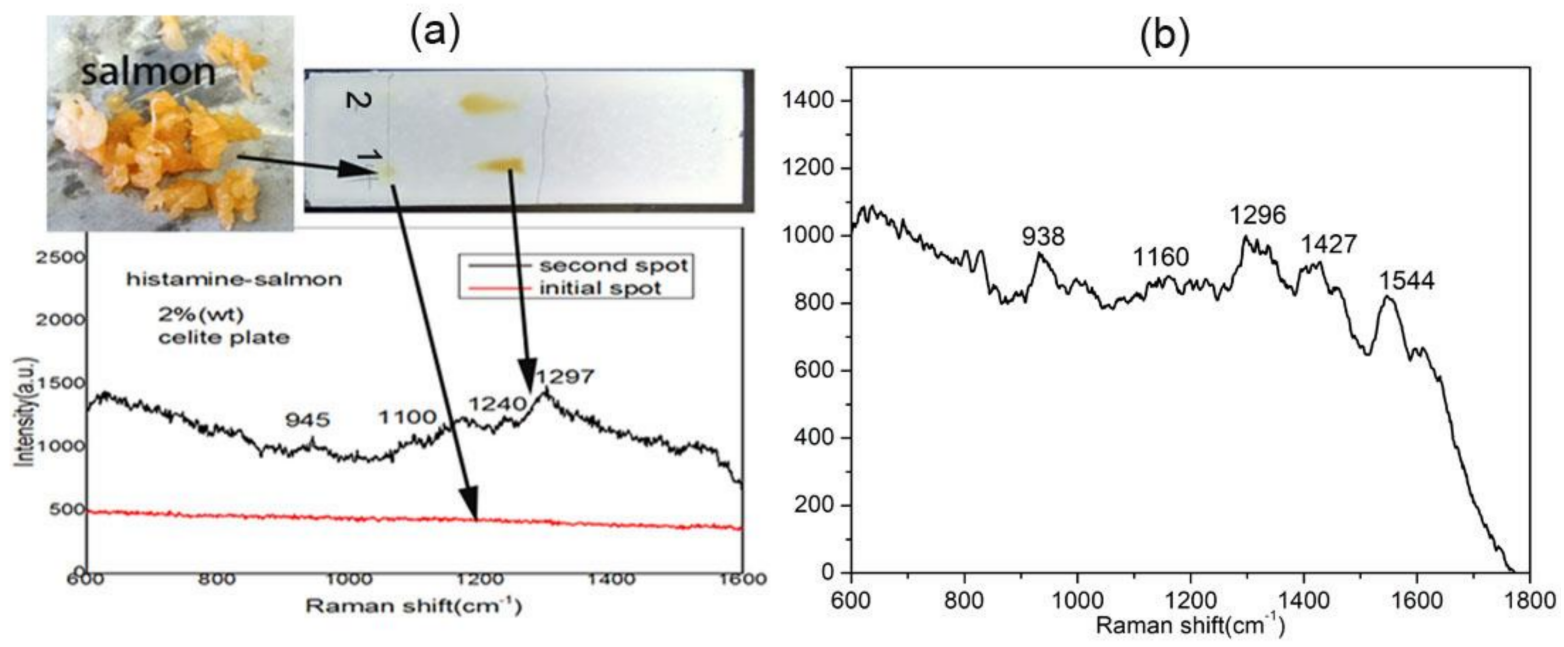
On-chip sensing of histamine from salmon (**a**) and 500 ppm of pure histamine solution (**b**).

Histamine is a kind of chemical compound with a low molecular weight of 111.15 Da, which was first described in 1910 by Henry H. Dale and P. P. Laidlaw [37]. Histamine has been studied in relation to pathological processes, such as acute allergies, inflammation of immune system, and so on. Histamine is colorless and odorless; thus, it is difficult to detect a high histamine content in fish without obvious changes in the appearance or smell of the fish. In our test, the salmon meat sample is chopped by scissors, and then, pure histamine (2% wt. in salmon) was mixed with the salmon as shown in Figure 6a. Then, 1 μL of the mixed sample was spotted onto the bottom of the diatomite plate. After development with an eluent (ammonia/ethanol = 1/1), the spot on the diatomite chip was visualized via iodine colorimetry. The control experiment was developed in parallel with a pure histamine solution on the same diatomite plate. The Raman spectra were collected after the Au NPs were casted onto different spots of the TLC chip. Figure 6b shows the SERS spectra of the pure histamine solution after TLC separation, which were consistent with Lin’s report on histamine Raman analysis [38]. The peak at 938 cm^−1^ is assigned to the vibration of ring, 1160 cm^−1^ is assigned to imidazole C-H in-plane bending, and the peak located at 1296 cm^−1^ is assigned to the imidazole ring stretching of histamine. The same retention factor (Rf)—which equals the ratio of the distance migrated by the target analyte and the solvent on the chromatography plate—values for histamine on the diatomite chip were obtained from these two samples as shown in Figure 6a. Furthermore, the SERS spectra of histamine separated from salmon is similar to that from pure histamine. The Rf values and SERS spectra proved that the diatomite plate could conduct separation and detection of histamine from the fish sample.

Seafood is one of main source of protein in many diets around the world. However, histamine can be produced in fish by bacterial enzymatic decarboxylation of histidine [39]. Fresh fish usually contain negligible amounts of histamine. However, several fish species, such as tunas, mackerel, mahi, bonito, bluefish, and sardines, which have high contents of histidine in their muscles, are more likely to contain histamine due to the bacterial enzymatic activity if the fish is not properly stored before consumption.

**Figure 7:**
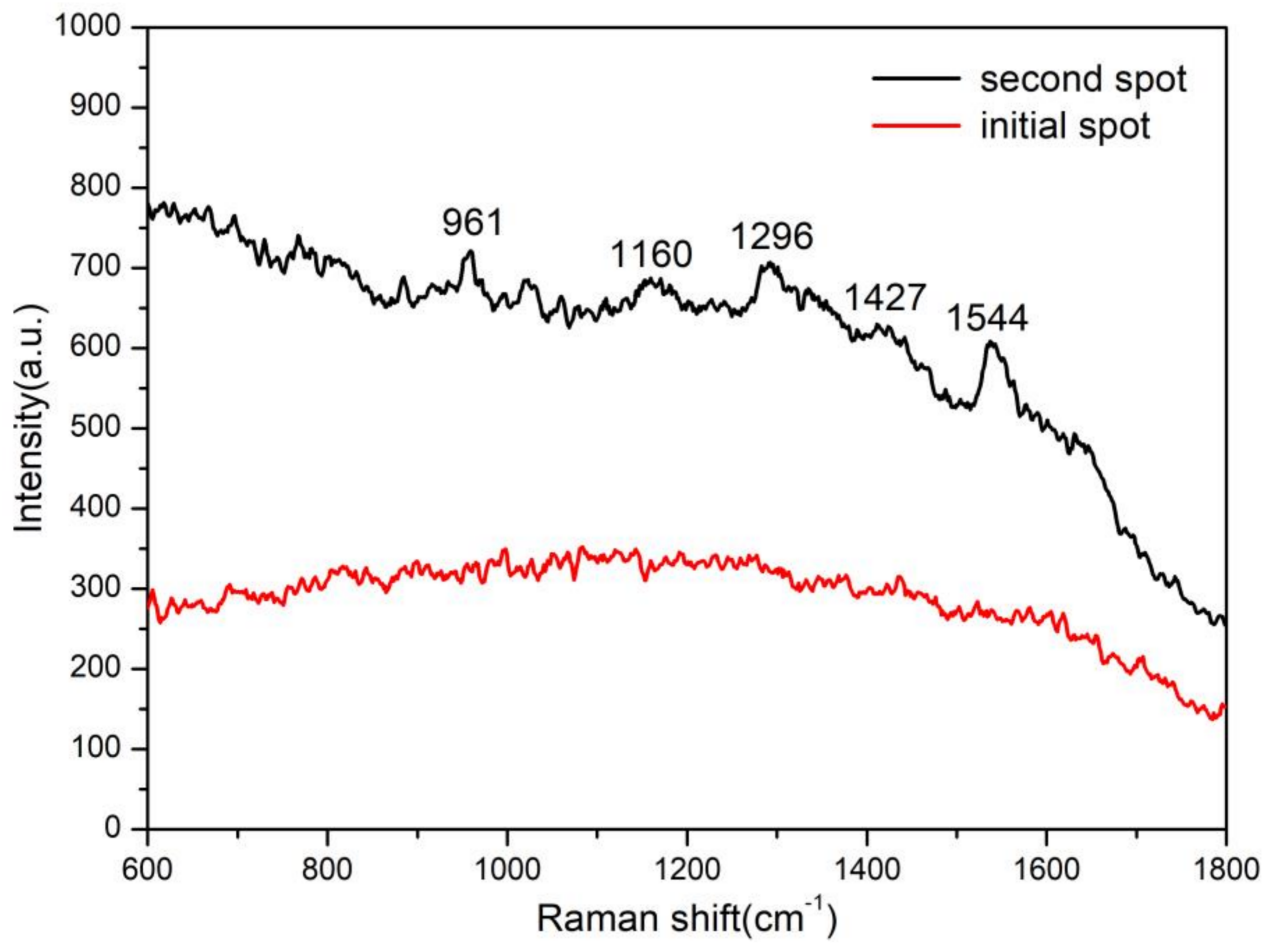
On-chip monitoring of the real fish sample (decomposed Tuna).

The diatomite TLC chip was also used for monitoring histamine in decomposed tuna. The blended sample of decomposed tuna mince was spotted onto the diatomite TLC chip. SERS measurement at the initial dropping spot results in no meaningful spectra because of various interferences from the tuna meat. After separation, the SERS spectra are shown in Figure 7. The feature SERS peaks of histamine were observed obviously, which indicated the existence of histamine in the decomposed tuna. The estimated concentration of histamine in the decomposed tuna was nearly 150 ppm as compared to the spectra intensity of the standard histamine spectra (Figure 6b).

## 4. Conclusions

In this research, a simple and cheap device was proposed to isolate and identify harmful ingredients from mixtures by fabricating a diatomite chip in the TLC-SERS method. The measurement results of the diatomite chip show almost 10 times the increment of SERS signal intensity and better uniformity as compared to normal TLC chips. These improvements resulted from the thinner stationary phase layer and the photonic-crystal features of diatomite. This device detected histamine from salmon without sample preprocessing. Furthermore, we detected histamine from decomposed tuna by using TLC-SERS for the first time as a food safety sensor. This on-chip device using diatomite photonic crystal biosilica can be applied as a cheap and robust biosensor for monitoring other kinds of harmful ingredients in real food samples to ensure food quality.

## Supplementary Materials

The following are available online at http://www.mdpi.com/1996-1944/11/4/539/s1: Figure S1: The UV-VIS absorption spectrum of the prepared Au colloids; Figure S2: Microscopic image of the cross-section of the commercial silica gel TLC chip.
