# Supplementary material for: Diatomite Photonic Crystals for Facile On-Chip Chromatography and Sensing of Harmful Ingredients from Food
Source: Materials (Basel). 2018 Mar 31;11(4):539. doi: 10.3390/ma11040539 (PMC5951423; doi:10.3390/ma11040539)
Supplement: Supplementary file 1 [file materials-11-00539-s001.pdf]

materials

MDPI

Supplementary

Diatomite Photonic Crystals for Facile On-Chip Chromatography and Sensing of Harmful Ingredients from Food

Xianming Kong [1], Qian Yu [1,*], Erwen Li [2], Rui Wang [1], Qing Liu [3] and Alan Wang [2,*]

1 College of Chemistry, Chemical Engineering and Environment Engineering, Liaoning Shihua University, Fushun 113001, Liaoning, China; xmkong@lnpu.edu.cn (X.K.); rwang@lnpu.edu.cn (R.W.)
2 School of Electrical Engineering and Computer Science, Oregon State University, Corvallis, OR 97331, USA; lie@oregonstate.edu
3 Key Laboratory of Low Carbon Energy and Chemical Engineering, College of Chemical and Environmental Engineering, Shandong University of Science and Technology, Qingdao 266590, Shandong, China; qliu@sdust.edu.cn
* Correspondence: qyu@lnpu.edu.cn (Q.Y.); wang@eecs.oregonstate.edu (A.W.); Tel.: +1-541-737-4247 (A.W.)

Received: 6 March 2018; Accepted: 29 March 2018; Published: 31 March 2018

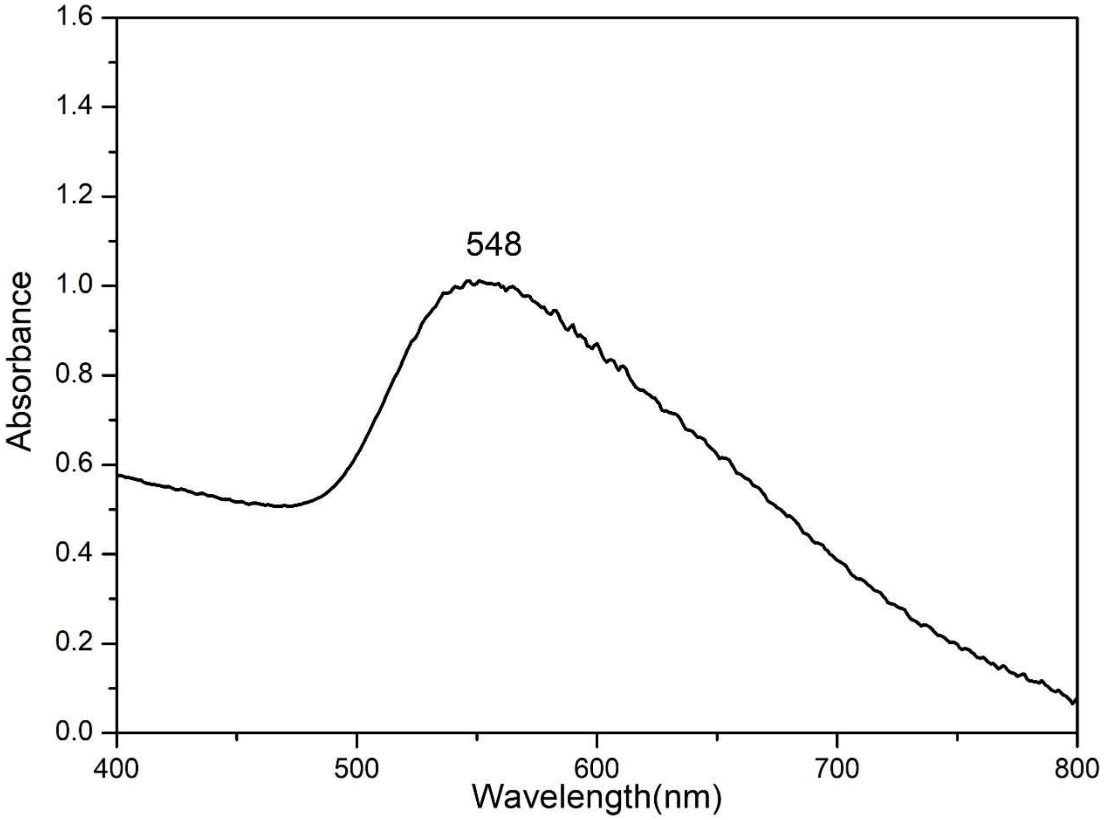

Figure S1. The UV-VIS absorption spectrum of the prepared Au colloids.

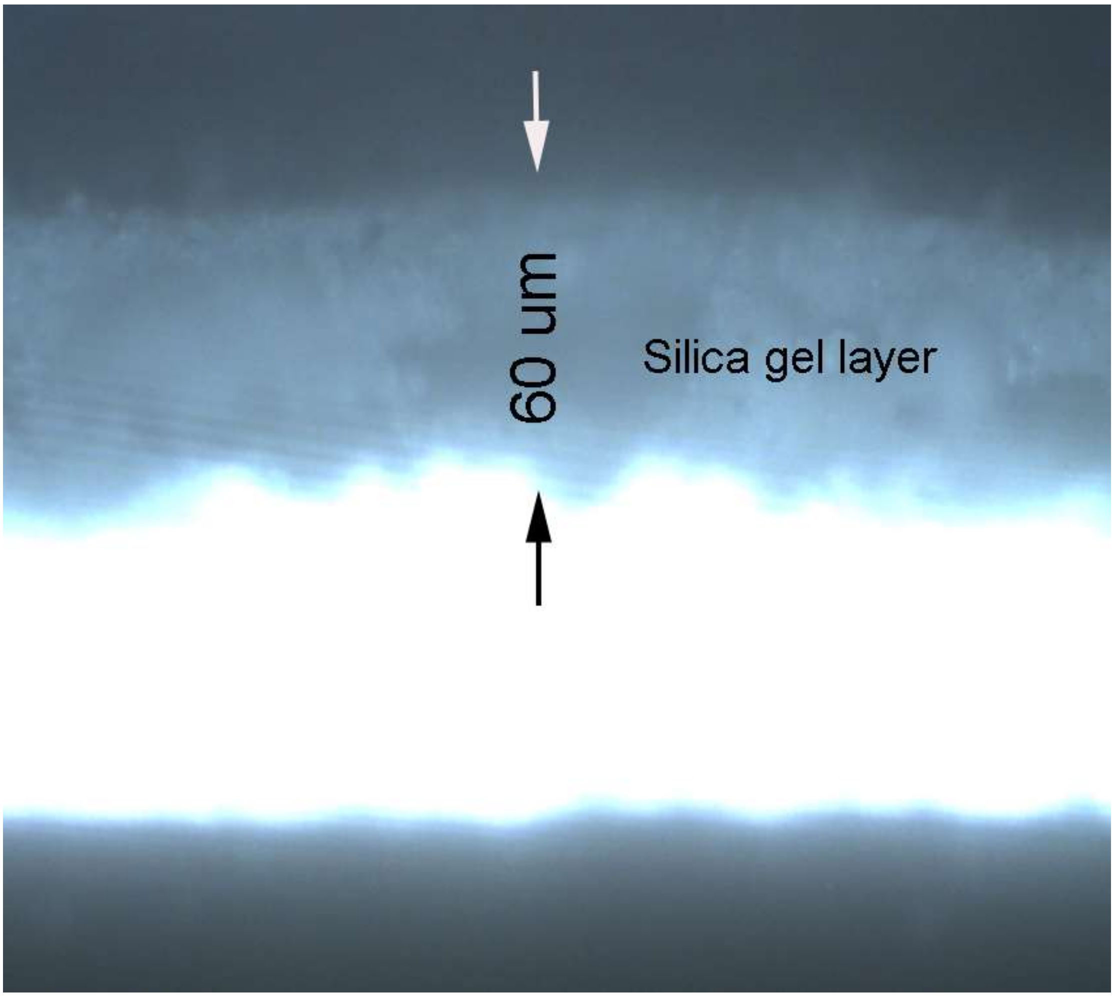

Figure S2. Microscopic image of the cross-section of the commercial silica gel TLC chip.
